# Ternary selenides A_2_Sb_4_Se_8_ (A = K, Rb and Cs) as an n-type thermoelectric material with high power factor and low lattice thermal conductivity: importance of the conformationally flexible Sb–Se–Se–Sb bridges†

**DOI:** 10.1039/d0ra01751e

**Published:** 2020-04-07

**Authors:** Changhoon Lee, Sujee Kim, Won-Joon Son, Ji-Hoon Shim, Myung-Hwan Whangbo

**Affiliations:** Department of Chemistry, Pohang University of Science and Technology Pohang 37673 Korea jhshim@postech.ac.kr; Division of Advanced Nuclear Engineering, Pohang University of Science and Technology Pohang 37673 Korea; Samsung Advanced Institute of Technology (SAIT), Samsung Electronics 130 Samsung-ro, Yeongtong-gu Suwon 16678 Korea; Department of Chemistry, North Carolina State University, Raleigh NC 27695-8204 USA whangbo@ncsu.edu; State Key Laboratory of Structural Chemistry, Fujian Institute of Research on the Structure of Matter (FJIRSM), Chinese Academy of Sciences (CAS) Fuzhou 350002 China; State Key Laboratory of Crystal Materials, Shandong University Jinan 250100 China

## Abstract

We investigated the thermoelectric properties of the layered ternary selenides A_2_Sb_4_Se_8_ (A = K, Rb and Cs) and the lattice thermal conductivity of K_2_Sb_4_Se_8_ on the basis of DFT calculations, to find that these selenides are a high-performance n-type thermoelectric material. The Seebeck coefficients and power factors calculated for the electron carriers of A_2_Sb_4_Se_8_ (A = K, Rb and Cs) are greater than those of the well-known thermoelectric materials Bi_2_Te_3_ and PbTe. The lattice thermal conductivity *κ*_latt_ of K_2_Sb_4_Se_8_ is comparable to that of PbTe, well-known for its low lattice thermal conductivity. In terms of both electronic and phonon structures, the structural parts of the A_2_Sb_4_Se_8_ (A = K, Rb and Cs) phases crucial for their thermoelectric properties are the conformationally-flexible Sb–Se–Se–Sb bridges that interlink between their structurally rigid units.

## Abstract 2

The ternary selenides A_2_Sb_4_Se_8_ (A = K, Rb, Cs) are predicted to be a high-performance n-type thermoelectric material, and the conformationally-flexible Sb–Se(2)–Se(2)–Sb bridges are crucial in determining the thermoelectric properties of A_2_Sb_4_Se_8_.

## Graphical abstract

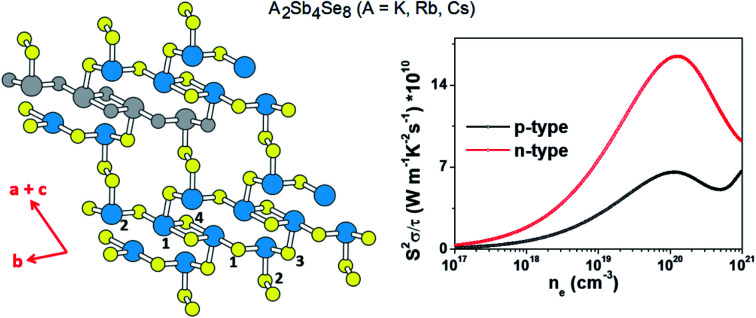

## Introduction

Thermoelectricity enables a direct conversion between thermal and electrical energies, so materials exhibiting thermoelectricity have received much attention for power generation and cooling.^1,2^ In particular, thermoelectric power generation is potentially important for waste heat collection and efficient energy utilization, although its application is limited due to the low efficiency of thermoelectric devices. The efficiency of a thermoelectric material at a given temperature *T* depends on the figure of merit, *ZT* = *S*^2^*σT*/*κ*, where *σ*, *κ* and *S* are the electrical conductivity, the thermal conductivity and the Seebeck coefficient, respectively.^3^ The amount of power generated by a thermoelectric material is related to the power factor, *S*^2^*σ*, which is the major element governing the magnitude of *ZT*.^4^ Another element raising the *ZT* is a low thermal conductivity *κ*, which is composed of the electric thermal conductivity (*κ*_el_) and the lattice thermal conductivity (*κ*_latt_), that is, *κ* = *κ*_el_ + *κ*_latt_. *κ*_el_ is directly proportional to the carrier concentration, *n*, because *κ*_el_ ∝ *σ via* the Wiedemann–Franz law, *κ*_el_ = *LσT*,^5^ where *L* is Lorenz number, and because *σ* ∝ *n*. In general, a high figure of merit *ZT* requires a compromise between the carrier concentration and the thermal conductivity.^6,7^ In terms of the carrier densities affecting the thermoelectric properties of a material, the electronic states lying close to its conduction band minimum (CBM) and valence band maximum (VBM) (*i.e.*, those typically within ∼0.5 eV of the CBM or the VBM) are found to be crucial.^8^ From the viewpoint of understanding the structure–property relationship governing the thermoelectric properties of a material, it is important to find which structural part of the material is largely responsible for those states close to the CBM and VBM.

**Fig. 1:**
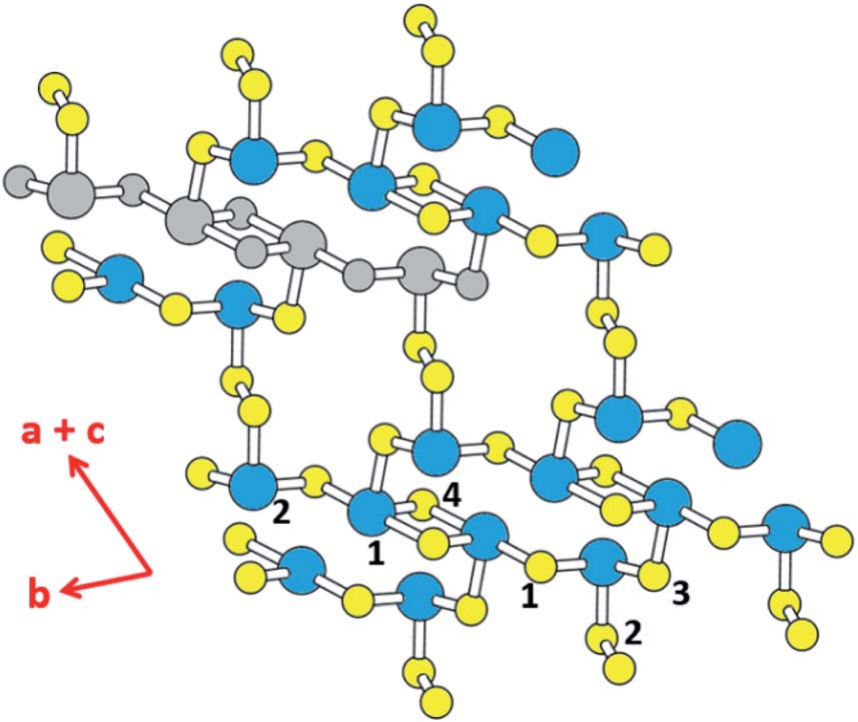
Schematic diagram showing the structure of the Sb_4_Se_8_ layer of K_2_Sb_4_Se_8_. The large and small circles represent the Sb and Se atoms, respectively. The Sb(1) and Sb(2) atoms are represented by blue circles, and Se(1)–Se(4) atoms by yellow circles, except for one Sb_4_Se_6_ unit. The latter is indicated by grey circles for both Sb and Se, for clarity. The Sb_4_Se_8_ layer is composed of 12- and 14-membered rings that are cross-linked. The cross-linked 12-membered rings form the Sb_4_Se_6_ chains running along the *b*-direction. The Sb_4_Se_6_ chains are linked by Se_2_ dimers to form 14-membered rings that are cross-linked. It should be noted that the *b*- and (*a* + *c*)-crystallographic directions of K_2_Sb_4_Se_8_ are the same as those of Rb_2_Sb_4_Se_8_, but correspond to the *a*- and (*b*–*c*)-directions of Cs_2_Sb_4_Se_8_, respectively.

The binary tellurides Bi_2_Te_3_ (ref. 9*a*) and PbTe^9*b*^ are well-known efficient thermoelectric materials. It is of interest and importance to search for a potential high-performance thermoelectric material that can be as efficient as Bi_2_Te_3_ and PbTe. In the present work we carry out DFT calculations to explore the thermoelectric properties of the layered ternary selenide K_2_Sb_4_Se_8_ (ref. 10) as well as its isostructural phases Rb_2_Sb_4_Se_8_ (ref. 11*a*) and Cs_2_Sb_4_Se_8_.^11*b*^ As depicted in Fig. 1, K_2_Sb_4_Se_8_ consists of Sb_4_Se_8_ layers separated by K^+^, and each layer is made up of Sb(1)Se_4_ seesaw units, Sb(2)Se_3_ pyramids, and Se_2_ dimers. There are two types of cross-linked rings in each Sb_4_Se_8_ layer; one is the 12-membered ring made up of two Sb(1)_2_Se_6_ and two Sb(2)Se_3_ units, and the other is the 14-membered ring made up of four Sb(2)Se_3_, two Se(2)_2_ and two Sb(1)Se_4_ seesaw units. From the viewpoint of understanding the lattice thermal conductivity of K_2_Sb_4_Se_8_, it is more convenient to consider each Sb_4_Se_8_ layer in terms of the “Sb_4_Se_6_” chains made up of cross-linked 12-membered rings, which run along the *b*-direction. For clarity, one Sb_4_Se_6_ repeat unit of this chain is colored in grey in Fig. 1. When these Sb_4_Se_6_ chains are bridged by the Se(2)_2_ dimers to form 14-membered rings between the Sb_4_Se_6_ chains, we obtain the Sb_4_Se_8_ layer.

Since the Sb–Se(2)–Se(2)–Sb bridges have a high conformational flexibility (namely, a soft potential energy curve with respect to a small variation in the dihedral angle ∠Sb–Se(2)–Se(2)–Sb), it is expected that the Sb_4_Se_8_ layer is more rigid along the Sb_4_Se_6_ chain direction (*i.e.*, along the *b*-direction for K_2_Sb_4_Se_8_) than along the interchain direction [*i.e.*, along the (*a* + *c*)-direction for K_2_Sb_4_Se_8_]. In addition, the K_2_Sb_4_Se_8_ crystal should be least rigid along the interlayer direction because there is no covalent bonding between adjacent Sb_4_Se_8_ layers. In this low-dimensional structure of K_2_Sb_4_Se_8_ as determined by X-ray diffraction, the unit cell parameters are large so that the lattice thermal conductivity would be low with short mean path for acoustic phonons.^12^ Thus, it is worthwhile investigate the possibility that K_2_Sb_4_Se_8_ as well as its two isostructural selenides, Rb_2_Sb_4_Se_8_ and Cs_2_Sb_4_Se_8_, are a high-performance thermoelectric material. In the present work, we examine the Seebeck coefficients and power factors of A_2_Sb_4_Se_8_ (A = K, Rb, Cs) and the lattice thermal conductivity of K_2_Sb_4_Se_8_ on the basis of DFT calculations, to predict that the ternary selenides A_2_Sb_4_Se_8_ (A = K, Rb, Cs) are a high-performance thermoelectric material comparable in efficiency to, or better than, the well-known thermoelectric materials Bi_2_Te_3_ and PbTe.

## Details of calculations

Our DFT calculations employed the frozen-core projector augmented wave (PAW) method^13^ encoded in the Vienna *ab initio* simulation package (VASP),^14^ with the generalized-gradient approximation (GGA)^15^ of Perdew, Burke and Ernzerhof (PBE) for the exchange–correlation functional and the plane-wave-cut-off energy of 450 eV. The BoltzTrap code^16^ was used to calculate the thermoelectric properties of all K_2_Sb_4_Se_8_ systems, which solves the semi-classical Boltzmann equation using the rigid band approach.^17,18^ This method has been successful in calculating the transport properties and predicting the optimal doping levels for thermoelectric materials.^17–20^ To ensure the convergence of the calculated thermoelectric properties, the irreducible Brillouin zone was sampled by a set of 2000 *k*-points. The BoltzTrap code allows one to calculate the electrical conductivity *σ*, the Seebeck coefficient *S*, and the power factor *S*^2^*σ*/*τ* under the assumption that the electron momentum relaxation time *τ* is independent of energy. We employ the latter assumption, because there is currently no information about the *τ* of A_2_Sb_4_Se_8_. The power factors calculated under this assumption are greater than the experimental values,^21^ but this overestimation arises in part from the fact that the band gaps are under estimated by DFT calculations. We note that the thermoelectric properties of numerous systems have been explained by using this assumption.^22^ We simulate the phonon behavior of K_2_Sb_4_Se_8_ by using the frozen phonon method implemented in Phono3py^23^ to investigate the lattice thermal conductivity.

## Electronic structures

**Fig. 2:**
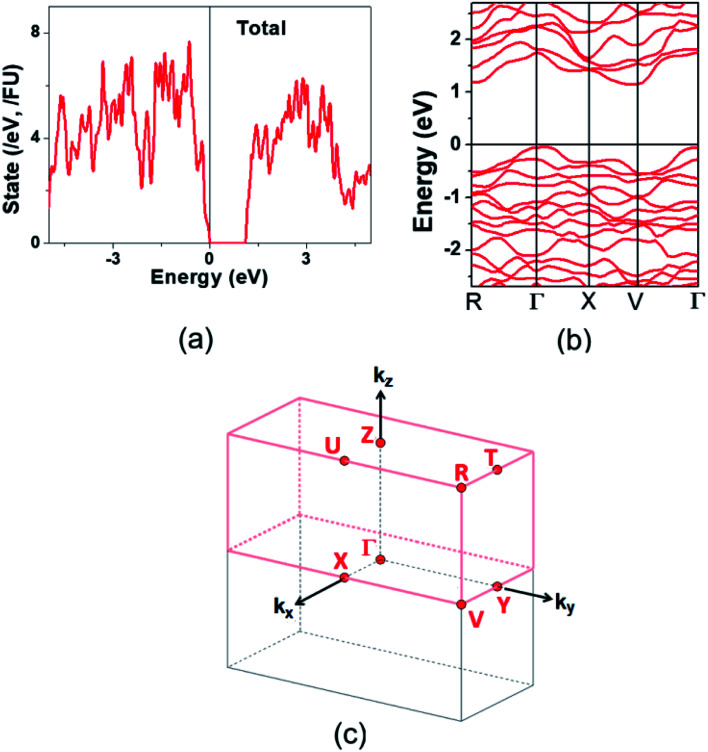
Electronic structure calculated for K_2_Sb_4_Se_8_: (a) Total DOS plot. (b) Band dispersion relations. (c) First Brillouin zone associated with a crystal lattice of space group *P*1̄.

**Fig. 3:**
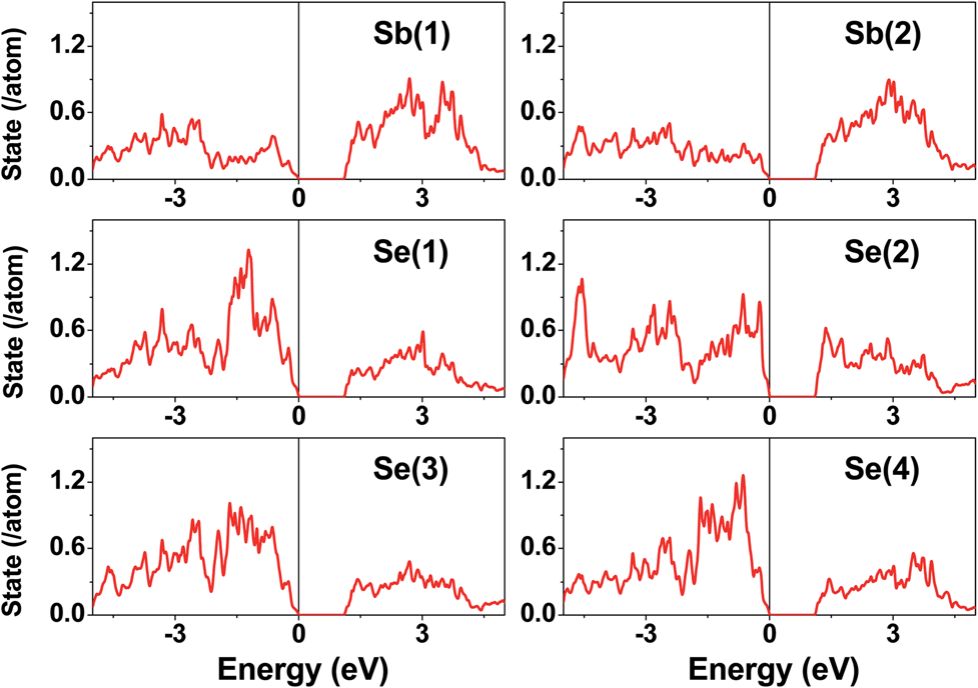
PDOS plots calculated for the Sb(1), Sb(2), Se(1), Se(2), Se(3), and Se(4) atoms of K_2_Sb_4_Se_8_.

The electronic structure calculated for K_2_Sb_4_Se_8_ is presented in terms of the total density of states (DOS) in Fig. 2a, and in terms of band dispersion relations in Fig. 2b along several wave vector directions of the first Brillouin zone (Fig. 2c). K_2_Sb_4_Se_8_ has a band gap of ∼1.0 eV. The projected DOS (PDOS) calculated for the Sb and Se atoms of K_2_Sb_4_Se_8_ are presented in Fig. 3. The PDOS plots for the Se(1), Se(3) and Se(4) are very similar but differ considerably from that for Se(2). In the occupied states of the PDOS, the Se(1), Se(3), and Se(4) each show two band blocks, which represent the bonding states between Sb and Se and the nonbonding states of Se. In contrast, the Se(2) shows roughly three band blocks, which arise from the Se(2)–Se(2) bonding states in addition to the Se–Sb bonding and the Se nonbonding states.

This reflects that the formal oxidation state of Se(1), Se(3), and Se(4) atoms is −2 whereas that of the Se(2) is −1. At the CBM and VBM, the Se(2) contributes more strongly than does the Se(1), Se(3) or Se(4) atom, and than does the Sb(1) or Sb(2) atom. Since the thermoelectric properties of a material are mainly influenced by the electronic states lying very close to its CBM and VBM (*i.e.*, typically those within ∼0.5 eV from the VBM and CBM),^8^ the thermoelectric properties of K_2_Sb_4_Se_8_ should be strongly influenced by the electronic structures associated with the Se(2)_2_ dimer units. The total DOS plots of Rb_2_Sb_4_Se_8_ and Cs_2_Sb_4_Se_8_, presented in Fig. S1 in the ESI,† are very similar to that of K_2_Sb_4_Se_8_ presented in Fig. 2.

## Thermoelectric properties

We calculate the Seebeck coefficients of A_2_Sb_4_Se_8_ (A = K, Rb, Cs) at 300 K as a function of the chemical potential *μ*. In terms of the rigid band approximation, the electron density can be introduced into A_2_Sb_4_Se_8_ by raising the Fermi level *E*_f_ from the CBM, so the chemical potential *μ* defined as *μ* = *E*_f_ − CBM, with the electron density *n*(*μ*) given by
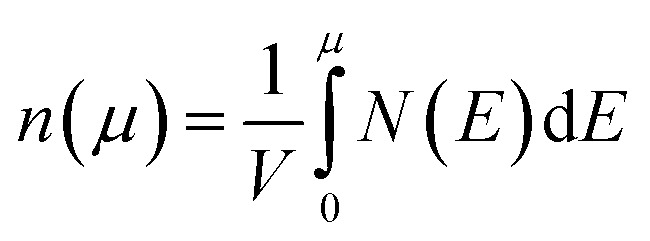
where *V* is the unit cell volume, and *N*(*E*) the total DOS at energy *E*. Similarly, the hole density can be introduced into A_2_Sb_4_Se_8_ by lowering the Fermi level *E*_f_ from the VBM, so the chemical potential *μ* is defined as *μ* = VBM − *E*_f_, with the hole density *p*(*μ*) given by
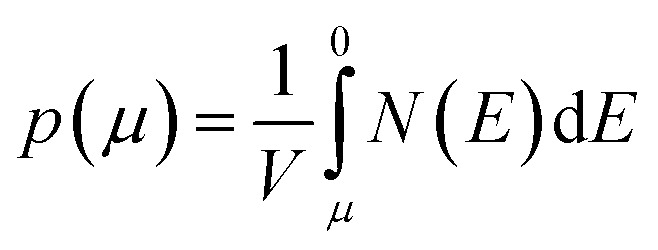


**Fig. 4:**
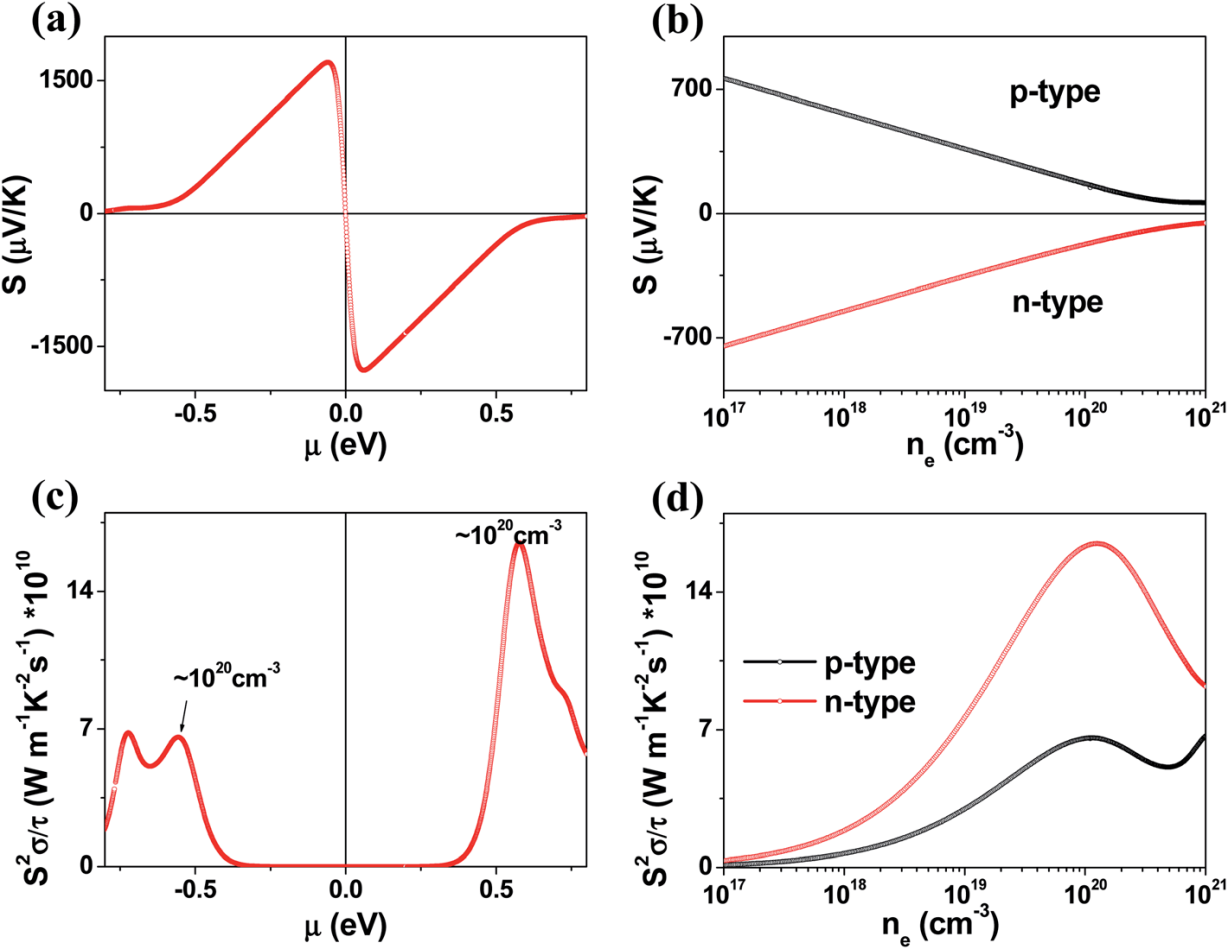
Thermodynamic properties of K_2_Sb_4_Se_8_ calculated at 300 K: (a) Seebeck coefficient S as function of the chemical potential *μ*. (b) Seebeck coefficient *S* as function of the carrier density *n*. (c) Power factor *S*^2^*σ*/*τ* as function of the chemical potential *μ*. (d) Power factor *S*^2^*σ*/*τ* as function of the carrier density *n*.

The *μ*-dependence of the Seebeck coefficients *S* calculated for K_2_Sb_4_Se_8_ (Fig. 4a) shows two peaks at *μ* ≈ ±0.061 eV with *S* ≈ ±1700 μV K^−1^. These values are considerably greater than those of the well-known thermoelectric materials Bi_2_Te_3_ (the calculated *S* ≈ ±250 μV K^−1^)^16^ and PbTe (the calculated *S* ≈ ±350 μV K^−1^).^20^ The dependence of *S* on the electron density and that on the hole density (Fig. 4b) reveals that the *S* decreases in magnitude steadily with increasing either the electron or the hole density. Under the assumption that the relaxation times *τ* is energy-independent, we calculate the power factor *S*^2^*σ*/*τ*. The calculated *S*^2^*σ*/*τ* is presented as a function of *μ* (Fig. 4c) and as a function of the carrier density (Fig. 4d). The power factor for the electron carriers is considerably greater than that for the hole carriers (*e.g.*, the n-type *S*^2^*σ*/*τ* is larger than the p-type *S*^2^*σ*/*τ* by a factor of ∼2). This reflects that the electrical conductivity for the electron carriers is much greater than that for the hole carriers. The Seebeck coefficients and the power factors calculated for Rb_2_Sb_4_Se_8_ and Cs_2_Sb_4_Se_8_ are summarized in Fig. S2, S3, and S5 in the ESI,† respectively. They are very similar to those described for K_2_Sb_4_Se_8_ described above.

We also investigated the temperature dependence of the Seebeck coefficients *S* and the power factor *S*^2^*σ*/*τ* for A_2_Sb_4_Se_8_ for A = K, Rb and Cs (Fig. S5 in the ESI†). In comparing these values of the three compounds, it is necessary to consider the possible uncertainties in their carrier densities. Thus, we calculate the Seebeck coefficients S and the power factor *S*^2^*σ*/*τ* for different carrier densities within the range between 1 × 10^19^ to 5 × 10^20^ cm^−3^. Fig. S5† reveals that, for both electron and hole carriers, the Seebeck coefficients S and the power factor *S*^2^*σ*/*τ* (in the upper and down panels, respectively) gradually increases with temperature. This is due to the increase in thermal energy. The power factor for the electron carriers is considerably greater than that for the hole carriers reflecting that the electrical conductivity for the electron carriers is much greater than that for the hole carriers.

The band dispersion relations calculated for K_2_Sb_4_Se_8_ (Fig. 2b) show that K_2_Sb_4_Se_8_ has an indirect band gap with the CBM at the M point and the VBM at the Γ point. The VBM has a hole valley at Γ, while the CBM has an electron valley and pseudo electron valley at M and R, respectively. The presence of flat band dispersion relations enhances the DOS and hence the carrier density. Since the CBM has more valleys than does the VBM, the carrier concentration should be enhanced near the CBM,^24^ so that the electrical conductivity is higher for the electrons than for the holes. This explains why the n-type power factor is considerably higher than the p-type power factor despite that the calculated Seebeck coefficients for the hole and electron carriers are similar. The band dispersion relations of Rb_2_Sb_4_Se_8_ and Cs_2_Sb_4_Se_8_, presented in Fig. S4 in the ESI,† are very similar to that of K_2_Sb_4_Se_8_ described above.

## Lattice thermal conductivity

**Fig. 5:**
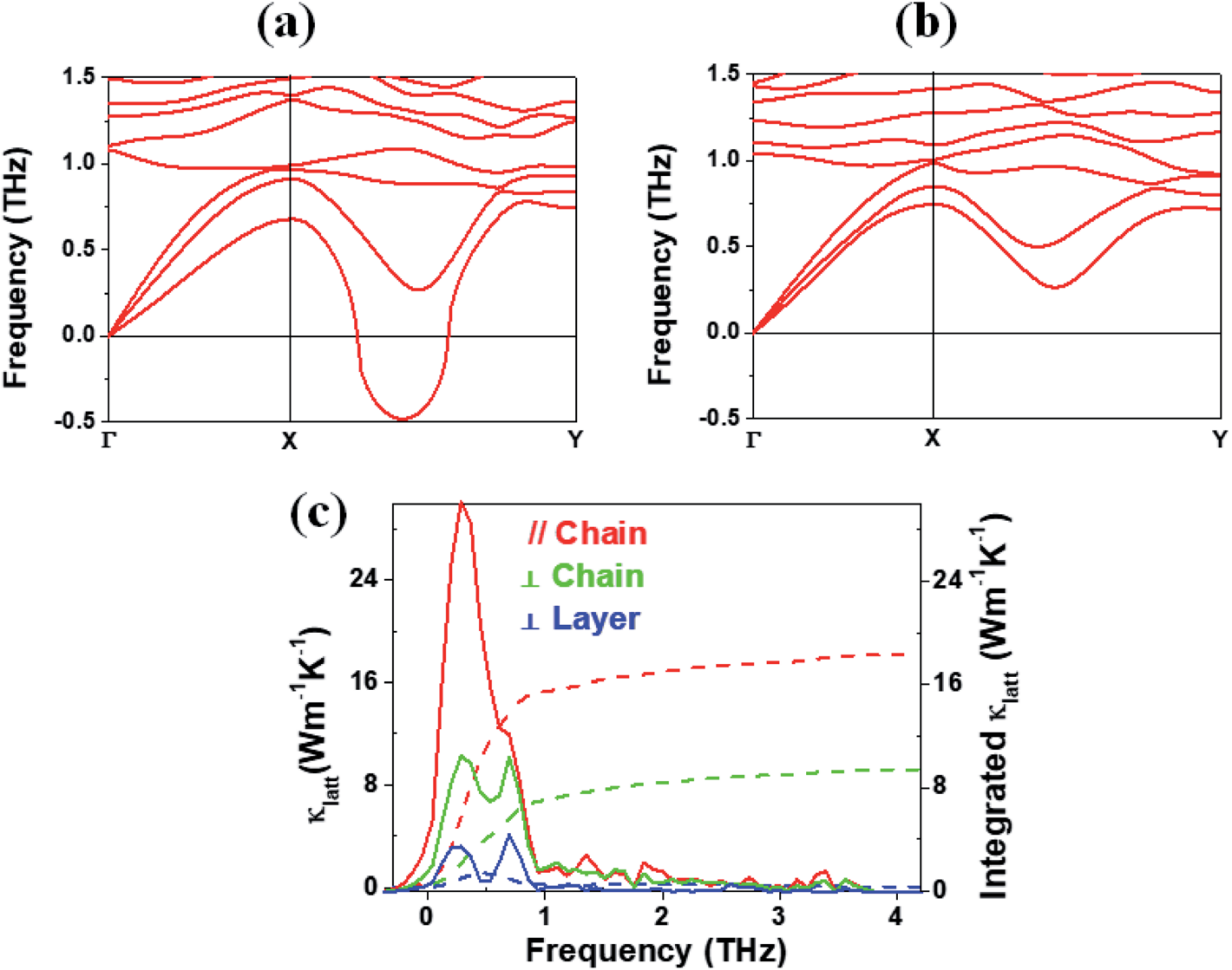
Phonon dispersion relations calculated for (a) the experimental and (b) optimized structures of K_2_Sb_4_Se_8_. (c) Lattice thermal conductivities *κ*_latt_ along the Sb_4_Se_6_ chain, along the interchain within the Sb_4_Se_8_ layer, and along the interlayer directions as a function of the frequency (solid lines). The integrated lattice thermal conductivities along the three directions are also shown (dashed lines).

In general, a low lattice thermal conductivity *κ*_latt_ is found for a low dimensional material with heavy elements and large unit cell parameters. K_2_Sb_4_Se_8_ is such a material. As already discussed (see Fig. 1), each Sb_4_Se_8_ layer of K_2_Sb_4_Se_8_ is more rigid along the Sb_4_Se_6_ chain direction (*i.e.*, the *b*-direction for K_2_Sb_4_Se_8_) than along the interchain direction within a Sb_4_Se_8_ layer [*i.e.*, the (*a* + *c*)-direction for K_2_Sb_4_Se_8_]. Thus, the thermal transport of K_2_Sb_4_Se_8_ should occur largely along the *b*-direction, and that along the (*a* + *c*)-direction should be weak, and so should be that along the (*a*–*c*)-direction (*i.e.*, the interlayer direction). We examine the phonon properties and the lattice thermal conductivity of K_2_Sb_4_Se_8_ using the Phono3py^23^ code combined with DFT calculations. The phonon dispersion relations calculated for K_2_Sb_4_Se_8_ using the experimental structure, shown in Fig. 5a, reveal the presence of an imaginary vibrational frequency. This indicates that the crystal structure as determined by X-ray diffraction measurements has a structural instability, suggesting the possibility that the real crystal structure has structural defects such as the charge density wave, local kinks, and fluctuations that are not detected by X-ray diffraction.^25^ This imaginary frequency disappears when the phonon dispersion relations are calculated for the structure of K_2_Sb_4_Se_8_ optimized by DFT calculations (Fig. 5b). Nevertheless, there exist low-frequency phonons, indicating the presence of a soft potential, which is associated most likely with the conformationally-flexible Sb–Se–Se–Sb bridges.

We calculate the lattice thermal conductivities by solving the linearized phonon Boltzmann equation with the single-mode relaxation time approximation, results of which are presented in Fig. 5c. As expected from the crystal structure of K_2_Sb_4_Se_8_, the thermal conductivities decrease in the order, the Sb_4_Se_6_ chain direction > the interchain direction of the Sb_4_Se_8_ layer > the interlayer direction. The lattice thermal conductivity *κ*_latt_ of K_2_Sb_4_Se_8_ is estimated to be 5.32 W mK^−1^, which is comparable to that of PbTe (with calculated *κ*_latt_ ≈ 2 W mK^−1^),^26^ a well-known material for low lattice conductivity. The low lattice thermal conductivity of K_2_Sb_4_Se_8_ is attributed to a decreased mean free path resulting from the large unit cell parameters, the low dimensional structure, and the structural instability arising from the conformationally flexible Sb–Se(2)–Se(2)–Sb bridges. For each b–Se(2)–Se(2)–Sb bridge, the potential energy curve with respect to a small change in the ∠Sb–Se(2)–Se(2)–Sb dihedral angle should be soft. It is most likely that the soft motions of the crystal lattice involve the slipping of each Sb_4_Se_6_ chain against its neighboring Sb_4_Se_6_ chains within each Sb_4_Se_8_ layer. The Sb–Se(2)–Se(2)–Sb bridges would act as phonon scattering centers rather than phonon transmittance paths due to their conformational flexibility. The lattice thermal conductivities of Rb_2_Sb_4_Se_8_ and Cs_2_Sb_4_Se_8_ should be similar to that of K_2_Sb_4_Se_8_ because Rb_2_Sb_4_Se_8_ and Cs_2_Sb_4_Se_8_ are very similar to K_2_Sb_4_Se_8_ in atomic and electronic structures.

## Discussion

**Fig. 6:**
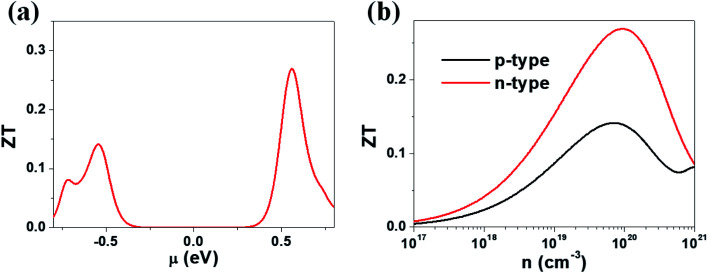
*ZT* values calculated for K_2_Sb_4_Se_8_ with *τ* ≈ 2 × 10^−14^ s: (a) the *μ*-dependence of the *ZT* using *κ*_latt_ = 5.32 W mK^−1^. (b) The carrier density dependence of the *ZT* using *κ*_latt_ = 5.32 W mK^−1^.

Our calculations suggest that the ternary selenides A_2_Sb_4_Se_8_ (A = K, Rb, Cs) have high Seebeck coefficients, high power factors for the electron carriers, and low thermal conductivities. We estimate the *ZT* value of K_2_Sb_4_Se_8_ at *T* = 300 K by using the calculated lattice thermal conductivity *κ*_latt_ of 5.32 W mK^−1^ and the relaxation time *τ* ≈ 2 × 10^−14^ s. In general, the relaxation time *τ* of conventional semiconductors is of the order of 10^−14^ s.^27^ For example, the relaxation times *τ* ≈ 2 × 10^−14^ and 3 × 10^−14^ s reproduce the experimental resistivities of Bi_2_Te_3_ and PbTe, respectively.^27,28^ The *ZT* values of K_2_Sb_4_Se_8_ estimated by using *τ* ≈ 2 × 10^−14^ s are presented in Fig. 6. The *μ*-dependence of the *ZT* (Fig. 6a) shows a peak at *μ* ≈ 0.56 eV with *ZT* ≈ 0.27, and another peak at *μ* ≈ −0.54 eV with *ZT* ≈ 0.14. The dependence of *ZT* on the carrier densities (Fig. 6b) reveals a peak at the electron carrier density of ∼10^20^ cm^−3^, which corresponds to the chemical potential *μ* ≈ 0.56 eV, and another peak at the hole carrier density of ∼7 × 10^19^ cm^−3^, which corresponds to the chemical potential *μ* ≈ −0.54 eV. The *ZT* of K_2_Sb_4_Se_8_ is greater for the electron carriers than for the hole carriers by a factor of ∼2. The calculated *ZT* values of K_2_Sb_4_Se_8_ are comparable to those estimated for Bi_2_Te_3_.^27*c*,29^

We note that the electronic states crucial for determining the n-type (p-type) Seebeck coefficients are those lying mainly within ∼0.5 eV from the CBM (VBM).^8^ Such states of A_2_Sb_4_Se_8_ (A = K, Rb, Cs) are represented largely by the Se(2) atoms forming the Sb–Se(2)–Se(2)–Sb bridges between two adjacent Sb_4_Se_6_ chains in each Sb_4_Se_8_ layer. Furthermore, the lattice thermal conductivity of A_2_Sb_4_Se_8_ (A = K, Rb, Cs) is expected to be lowered by the Sb–Se(2)–Se(2)–Sb bridges because they are conformational flexible as mentioned above. Thus, in terms of both electronic and vibrational factors, the Sb–Se(2)–Se(2)–Sb bridges play a crucial role in enhancing the thermoelectric properties of A_2_Sb_4_Se_8_ (A = K, Rb, Cs).

## Concluding remarks

Our work suggests that the layered ternary selenides A_2_Sb_4_Se_8_ (A = K, Rb, Cs) are a promising thermoelectric material comparable in efficiency to, or better than, the well-known thermoelectric materials such as Bi_2_Te_3_ and PbTe. The power factor and *ZT* values estimated for the electron carriers are considerably greater than those for the hole carriers by a factor of ∼2. Thus, the ternary selenides A_2_Sb_4_Se_8_ (A = K, Rb, Cs) are predicted to be a high-performance n-type thermoelectric material. The electronic and phonon structures associated with the Se(2)_2_ dimer units of the conformationally-flexible Sb–Se(2)–Se(2)–Sb bridges are crucial in determining the thermoelectric properties of A_2_Sb_4_Se_8_ (A = K, Rb, Cs).

## Conflicts of interest

There are no conflicts to declare.

## Supplementary Material

RA-010-D0RA01751E-s001

## References

[cit1] Nolas G. S. , Sharp J. and Goldsmid H., Thermoelectrics: Basic Principles and New Materials Developments, Springer, New York, 2001

[cit2] Rowe D. M. , Thermoelectrics Handbook: Macro to Nano, CRC Press, Boca Raton, 2006

[cit3] Minnich A. J., Dresselhaus M. S., Ren G. F., Chen G. (2009). Energy Environ. Sci..

[cit4] Baranowski L. L., Snyder G. J., Toberer E. S. (2013). J. Appl. Phys..

[cit5] Franz R., Wiedemann G. (1853). Ann. Phys..

[cit6] He J. Q., Kanatzidis M. G., Dravid V. P. (2013). Mater. Today.

[cit7] Zhao L. D., Dravid V. P., Kanatzidis M. G. (2014). Energy Environ. Sci..

[cit8] Lee C., Shim J. H., Whangbo M.-H. (2018). Inorg. Chem..

[cit9] Nakajima S. (1963). J. Phys. Chem. Solids.

[cit10] Sheldrick W. S., Wachhold M. (1998). Z. Kristallogr. NCS.

[cit11] Sheldrick W. S., Wachhold M. (1998). Z. Kristallogr. NCS.

[cit12] Sootsman J. R., Chung D. Y., Kanatzidis M. G. (2009). Angew. Chem., Int. Ed..

[cit13] Blöchl P. E. (1994). Phys. Rev. B: Condens. Matter Mater. Phys..

[cit14] Kresse G., Furthmüller J. (1996). Phys. Rev. B: Condens. Matter Mater. Phys..

[cit15] Perdew J. P., Burke K., Ernzerhof M. (1996). Phys. Rev. Lett..

[cit16] Madsen G. K. H., Singh D. (2006). Comput. Phys. Commun..

[cit17] Madsen G. K. H. (2006). J. Am. Chem. Soc..

[cit18] Scheidemante T. J., Ambrosch-Draxl C., Thonhauser T., Badding J. V., Sofo J. O. (2003). Phys. Rev. B: Condens. Matter Mater. Phys..

[cit19] Chaput L., Pécheur P., Tobola J., Scherrer H. (2005). Phys. Rev. B: Condens. Matter Mater. Phys..

[cit20] Singh D. J. (2010). Phys. Rev. B: Condens. Matter Mater. Phys..

[cit21] Chen W., Pöhls J.-H., Hautier G., Broberg D., Bajaj S., Aydemir U., Gibbs G. M., Zhu H., Asta M., Snyder G. J., Meredig B., White M. A., Perssonae K., Jain A. (2016). J. Mater. Chem. C.

[cit22] Lee C., Hong J., Whangbo M.-H., Shim J. H. (2013). Chem. Mater..

[cit23] Togo A., Chaput L., Tanaka I. (2015). Phys. Rev. B: Condens. Matter Mater. Phys..

[cit24] Hong J., Lee C., Park J.-S., Shim J. H. (2016). Phys. Rev. B: Condens. Matter Mater. Phys..

[cit25] Nielsen H. B., Ninomiya M. (2005). Prog. Theor. Phys..

[cit26] Tian Z., Garg J., Esfarjani K., Shiga T., Shiomi J., Chen G. (2012). Phys. Rev. B: Condens. Matter Mater. Phys..

[cit27] Jeon H.-W., Ha H.-P., Hyun D.-B., Shim J.-D. (1991). J. Phys. Chem. Solids.

[cit28] Ahmad S., Mahanti S. D. (2010). Phys. Rev. B: Condens. Matter Mater. Phys..

[cit29] Witting I. T., Chasapis T. C., Ricci F., Peters M., Heinz N. A., Hautier G., Snyder G. J. (2019). Adv. Electron. Mater..

