# Supplementary material for: Ternary selenides A2Sb4Se8 (A = K, Rb and Cs) as an n-type thermoelectric material with high power factor and low lattice thermal conductivity: importance of the conformationally flexible Sb–Se–Se–Sb bridges
Source: RSC Adv. 2020 Apr 7;10(24):14415–21. doi: 10.1039/d0ra01751e (PMC9051912; doi:10.1039/d0ra01751e)
Supplement: RA-010-D0RA01751E-s001 [file RA-010-D0RA01751E-s001.pdf]

Electronic Supplementary Material (ESI) for RSC Advances.
This journal is © The Royal Society of Chemistry 2020

Supporting Information

for

Ternary selenides $A_2Sb_4Se_8$ (A = K, Rb, Cs) as an n-type thermoelectric material with high power factor and low lattice thermal conductivity: Importance of the conformationally flexible Sb-Se-Se-Sb bridges

Changhoon Lee[1], Sujee Kim[1], Won-Joon Son[2], Ji-Hoon Shim[1]*, Myung-Hwan Whangbo[3-5]*

[1] Department of Chemistry, Pohang University of Science and Technology, Pohang, 37673, Korea

[2] Samsung Advanced Institute of Technology (SAIT), Samsung Electronics, 130 Samsung-ro, Yeongtong-gu, Suwon 16678, Korea

[3] Department of Chemistry, North Carolina State University, Raleigh, NC, 27695-8204, USA.
[4] State Key Laboratory of Structural Chemistry, Fujian Institute of Research on the Structure of Matter (FJIRSM), Chinese Academy of Sciences (CAS), Fuzhou, China, 350002

[5] State Key Laboratory of Crystal Materials, Shandong University, Jinan, China, 250100

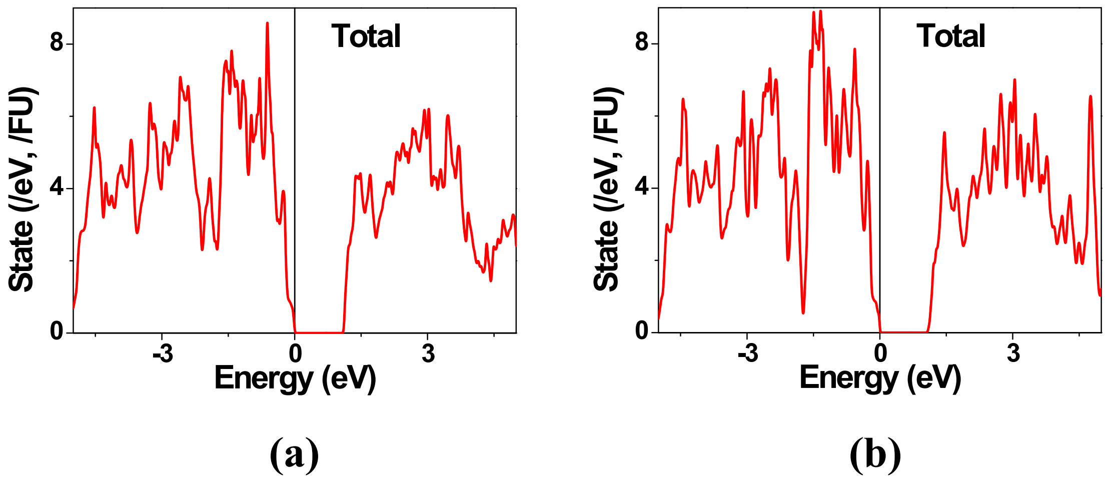

Figure S1. Total DOS plots calculated for (a) $Rb_2Sb_4Se_8$ and (b) $Cs_2Sb_4Se_8$.

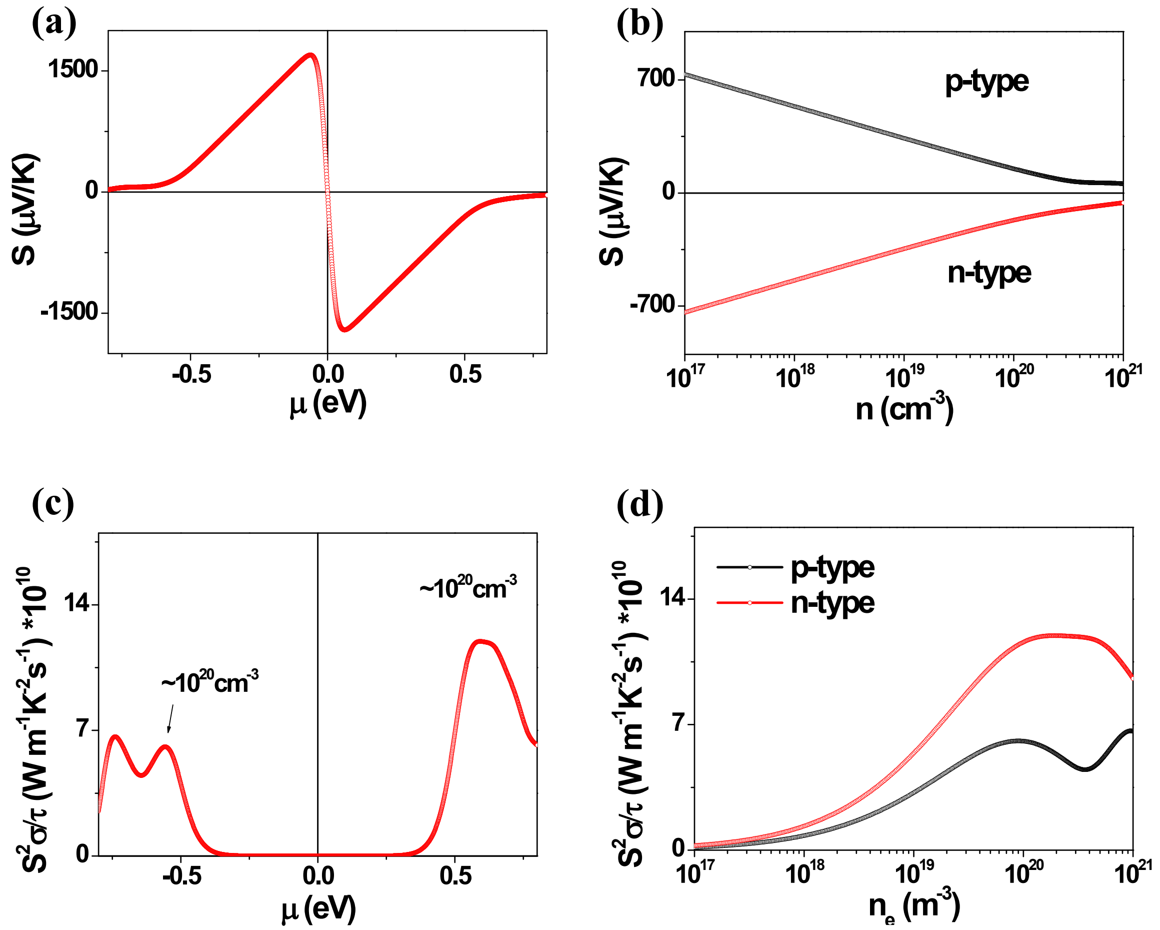

Figure S2. Thermodynamic properties of $Rb_2Sb_4Se_8$ calculated at 300 K: (a) Seebeck coefficient S as function of the chemical potential μ. (b) Seebeck coefficient S as function of the carrier density n. (c) Power factor $S^2\sigma/\tau$ as function of the chemical potential μ. (d) Power factor $S^2\sigma/\tau$ as function of the carrier density n.

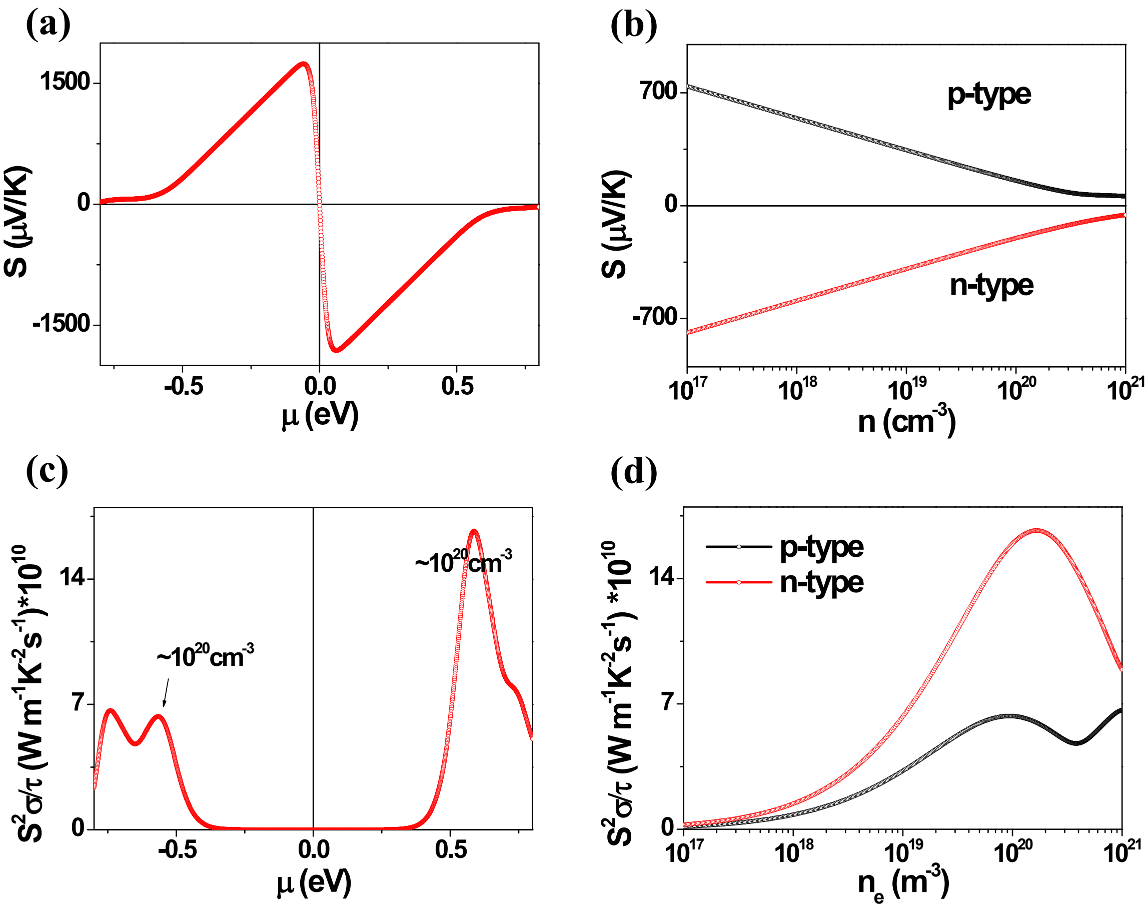

Figure S3. Thermodynamic properties of $Cs_2Sb_4Se_8$ calculated at 300 K: (a) Seebeck coefficient S as function of the chemical potential μ. (b) Seebeck coefficient S as function of the carrier density n. (c) Power factor $S^2\sigma/\tau$ as function of the chemical potential μ. (d) Power factor $S^2\sigma/\tau$ as function of the carrier density n.

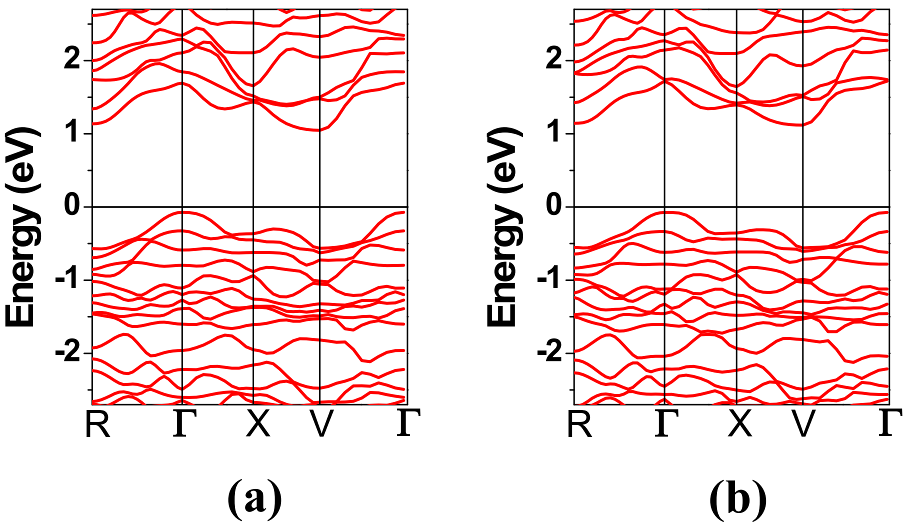

Figure S4. Band dispersion relations calculated for (a) $Rb_2Sb_4Se_8$ and (b) $Cs_2Sb_4Se_8$.

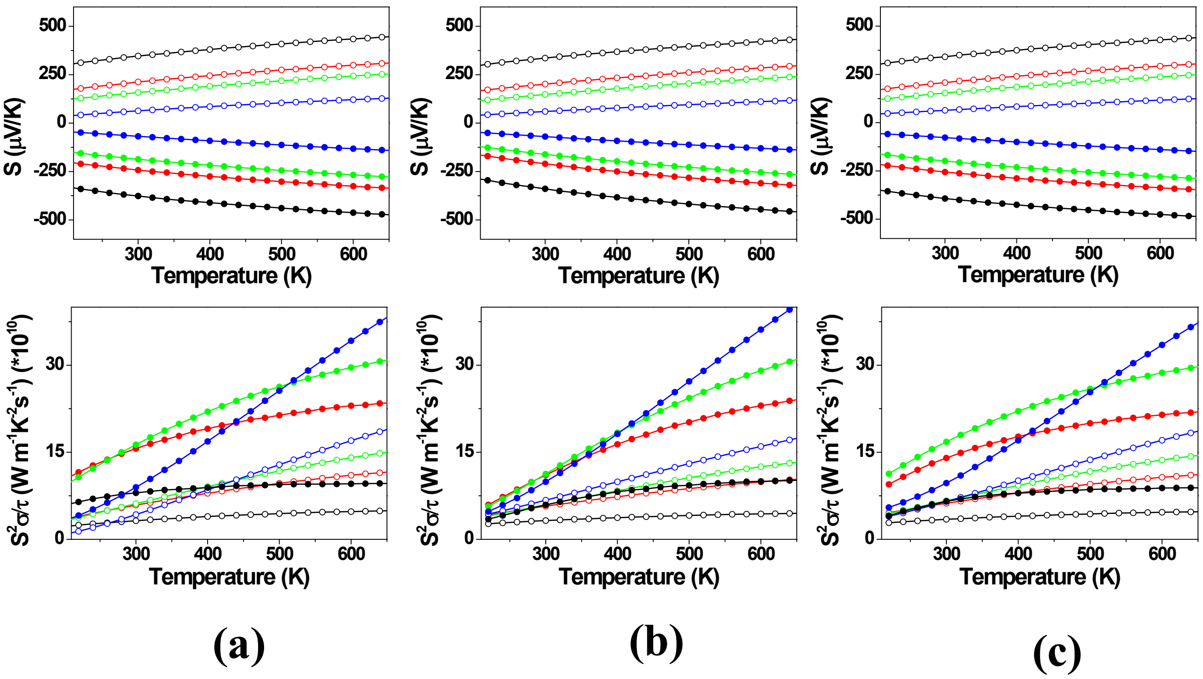

Figure S5. The temperature dependence of the Seebeck coefficients S (upper panels) and the power factor $S^2\sigma/\tau$ (down panels) plots calculated for (a) $K_2Sb_4Se_8$, (b) $Rb_2Sb_4Se_8$, and (c) $Cs_2Sb_4Se_8$. The black, red, green, and blue circles refer to carrier concentration 1×10^{19}, 5×10^{19}, 1×10^{20}, and 5×10^{20} cm^{-3}, respectively. The open and filled circles indicate hole- and electron-type carriers.
